# Supplementary material for: Intercontinental karyotype–environment parallelism supports a role for a chromosomal inversion in local adaptation in a seaweed fly
Source: Proc Biol Sci. 2018 Jun 20;285(1881):20180519. doi: 10.1098/rspb.2018.0519 (PMC6030540; doi:10.1098/rspb.2018.0519)
Supplement: Supplementary materials [file rspb20180519supp1.pdf]

Supplementary materials

Intercontinental karyotype-environment parallelism supports a role for a chromosomal inversion in local adaptation in a seaweed fly

Claire Mérot[1*], Emma L. Berdan[2], Charles Babin[1], Eric Normandeau[1], Maren Wellenreuther[3,4], Louis Bernatchez[1*]

[1]Département de biologie, IBIS, Université Laval, Canada

[2]Department of Marine Sciences, University of Gothenburg, Sweden.

[3]Department of Biology, Lund University, Lund, Sweden

[4]Plant & Food Research, Nelson, New Zealand

* Claire.Merot@gmail.com * Louis.Bernatchez@bio.ulaval.ca

1- Marker development 3
Methods: Development and validation of a DNA marker for the inversion 3
Fig S1: Electrophoresis of the *Adh* amplified locus digested by AluI and DraI. 4
Table S1: Position, primers and matching protein for the three coding regions sequenced 5
Table S2: Samples used for marker development and validation. 6
2- Inversion distribution & environmental variability in North America 8
Table S3: Environmental variables at sampled locations in North America 8
Table S4: Sample genotyped, frequencies and H-W proportions 9
Fig S2: Heterogeneity of inversion & karyotype frequencies between populations. 10
Fig S3: Heterogeneity of inversion frequency and karyotype proportions between populations. 10
Fig S4: Summary variables drawn by PCA on environmental variables 11
Fig S5: Association between environmental variables and inversion/karyotype frequencies 12
Table S5: Best models of binomial logistic regression and beta-regression explaining and predicting inversion or karyotype frequencies by a combination of environmental variables 13
Table S6: Dirichlet regression between karyotype composition and environmental variables. 15
Table S7: Best models of redundancy analysis including spatial autocorrelation 15
3- Size measurement and analysis 16
Fig S6: Wing length measurement 16
Fig S7: Size variation between populations in relation with local factors. 16
Fig S8: Difference between average male and female wing size at each population as a function of karyotype frequency. 17
4- Comparison between North American and European populations 18
Methods for comparison North America/Europe 18
Table S8: Environmental variables at locations sampled by Day *et al* (1983) (9) in Scandinavia 18
Results of the comparison North America/Europe 19
Fig S9: Comparing parallelism in the directionality of association between inversion/karyotype frequency and environmental variables 20
Fig S10: PCA on environmental variables in Scandinavia and association with inversion and karyotype frequencies 21
Table S9: Best models explaining and predicting inversion or karyotype frequencies in Scandinavia by a combination of environmental variables 21
Table S10: Best Dirichlet regression models explaining and predicting karyotype composition in Scandinavia by a combination of environmental variables transformed with PCA (cf Fig. S12) 21
References 22

1- Marker development

Methods: Development and validation of a DNA marker for the inversion

We used the previously-demonstrated association between the chromosome I inversion karyotype (α/β) and two common alleles (B/D) of the alcohol dehydrogenase (Adh) allozyme marker (1) to develop and validate an inversion-specific DNA marker.

First, we searched for the putative *Adh* locus by blasting the *Adh* protein sequences of *Drosophila* from GeneBank on the draft assembly of *Coelopa frigida* genome (M. Wellenreuther, unpublished). The two best matching scaffolds were aligned together using MAUVE and showed several well-aligned areas, within which three coding loci corresponding to dipteran proteins were identified using *Blastx* in the swissprot NCBI protein database (the alcohol dehydrogenase, *Adh,* a predicted 39S ribosomal protein, *Rib* and an arginine N-methyltransferase 1, *Met,* see Table S1).

Second, to amplify and sequence those three coding loci in *C. frigida*, we developed five sets of primers (Table S1). Initial sequencing was applied on 42 (*Adh*) and 31 (*Met, Rib*) flies from North America and Europe (details in Table S2). Genomic DNA was extracted from adult flies using a salt-extraction protocol (2) with a RNase A treatment (Quiagen) or a lysis protocol (3) . The same PCR assays were carried out for the three loci in a 25-µL final volume composed of 1 µL of gDNA, 0.4 µM forward primers, 0.4 µM reverse primers and either 10µL of QuantaBio MasterMix or a mix including: 1× Green GoTaq Flexi buffer, 0.625 units of GoTaq DNA polymerase (ProMega), 2.5mM of $MgCl_2$, 0.2 mM dNTP. The PCR amplification temperature profile consisted of an initial denaturation at 95 °C for 2 min followed by 35 cycles at 95°C for 45s; 55°C for 45 s and 72 °C for 1 min and a final elongation at 72 °C for 15 min. Sanger sequencing was performed with standard conditions at the Plate-forme d'Analyses Génomiques (Université Laval, Québec, Canada). Sequences of the three loci were cleaned for sequencing ambiguities and aligned using Geneious 9.1.7.

Third, a restriction enzyme procedure was developed to genotype two targeted SNPs in complete linkage with the *Adh* haplotypes (Fig.2), thus putatively associated with the inversion. On 42 samples, no other mutation than the targeted polymorphism was observed in the area to which the restriction enzymes linked. In a 10µL volume reaction, 8.5µL of PCR product was digested during 15min at 37°C with 5 units of the restriction enzyme AluI or DraI and 1x CutSmart Buffer (New England Biolabs). Digestion product was run on 2.5% agarose gel electrophoresis for 45min at 115V (Fig. S1).

To validate the association between the haplotype sequence and the inversion karyotype, 44 *Coelopa frigida* (4 αα, 17 αβ, 23 ββ, Table S2) were cut in two halves: the abdomen was used for allozyme characterization protocol as described in (4), which allow assessing the inversion karyotype, and DNA was isolated from the thorax for sequencing (12 samples) or genotyping with the restriction enzyme procedure (32 samples).

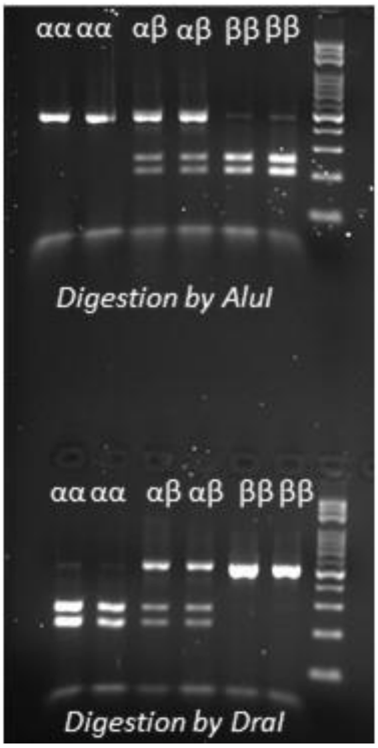

Fig S1: Electrophoresis of the *Adh* amplified locus digested by AluI and DraI.
The enzyme *Alu*I digests only the β haplotype and thus allows separating the αα individuals from the ones carrying the β haplotype. *Dra*I enzyme digests only the α haplotype and thus allows discriminating ββ individuals from the ones carrying the α haplotype

Table S1: Position, primers and matching protein for the three coding regions sequenced

Position on Scaffold 522	*Position on Scaffold 960*	*Locus name (in our study) & length*	*Primers used for amplification (F = forward, R= reverse)*	*Best matching protein sequence in NCBI database (and alternative)*
87 794 -> 88 528	88 -> 822	*Rib* 734 bp 650bp retained in analyses	>F88_Rib ACTTTCCCTTCACCGGTTCA >R822_Rib CCGACGCCAATGCTGAATTG	hypothetical protein FF38_01849 [Lucilia cuprina] KNC22169.1 (PREDICTED 39S ribosomal protein L37 [Drosophila elegans] XP_017083547.1)
90 028 -> 91 123	1498 -> 2593	*Met* 1095bp 953bp retained in analyses	>F86_Met TTACGCTCGTTGGGCTTCAT >F560_Met AGCCACTTGTCACGTGCATA >R818_Met CGCGGCAGGTTGTTATTGAC >R1181_Met GCCAACAATGTGCCCAACAA	arginine N-methyltransferase 1 [Ceratitis capitata] XP_004524313.1
99 574 -> 100 284	8753 -> 9463	*Adh* 710bp (508 bp) 476bp retained in analyses	>F67_Adh TCCATTTGGCATCAGCTCAC >F269_Adh TGACGTCTTGGTGAACGGAG >R777_Adh CTTCACAAAGTTCTGGCCGC	alcohol deshydrogenase [Sarcophaga peregrina] BAA09819.1

Table S2: Samples used for marker development and validation.

ID	Population	Coordinates	Continent	Sequenced at locus: Rib	Met	Adh	karyotype (allozyme)	karyotype (snp)
CE001	Cap Espoir	48.43086;-64.32778	North America	x	x	x		αα
CE002	Cap Espoir	48.43086;-64.32778	North America	x	x	x		αα
CE004	Cap Espoir	48.43086;-64.32778	North America	x	x	x		αα
CE006	Cap Espoir	48.43086;-64.32778	North America	x	x	x		αα
CE011	Cap Espoir	48.43086;-64.32778	North America	x	x			ββ
CE012	Cap Espoir	48.43086;-64.32778	North America	x	x	x		αβ
CE013	Cap Espoir	48.43086;-64.32778	North America	x	x	x		αβ
CE018	Cap Espoir	48.43086;-64.32778	North America	x	x	x		αα
CP002	Clam Point	44.72600; -62.91260	North America	x	x	x		αβ
CP004	Clam Point	44.72600; -62.91260	North America			x		αα
CP006	Clam Point	44.72600; -62.91260	North America	x	x	x		ββ
CP007	Clam Point	44.72600; -62.91260	North America			x		ββ
CP008	Clam Point	44.72600; -62.91260	North America	x	x	x		αβ
E00T2			Scandinavia	x	x	x		ββ
E00T3			Scandinavia	x	x	x		αα
E00T4			Scandinavia	x	x	x		ββ
E00T5			Scandinavia	x	x	x	ββ	ββ
E00T6			Scandinavia	x	x	x	ββ	ββ
E00T7			Scandinavia			x	ββ	ββ
ESF07	Smyggahuk	55.337224; 13.36013	Scandinavia	x	x	x	ββ	ββ
EYF05	Ystad	55.425; 13.77254	Scandinavia	x	x	x	ββ	ββ
EYF11	Ystad	55.425; 13.77254	Scandinavia	x	x	x	ββ	ββ
ME003	Métis	48.66408; -68.07221	North America	x	x	x		ββ
ME032	Métis	48.66408; -68.07221	North America			x		αβ
ME051	Métis	48.66408; -68.07221	North America	x	x	x		ββ
ME052	Métis	48.66408; -68.07221	North America			x		ββ
ME059	Métis	48.66408; -68.07221	North America			x		αβ
ME068	Métis	48.66408; -68.07221	North America			x		αβ
ME074	Métis	48.66408; -68.07221	North America	x	x	x		αβ
ME083	Métis	48.66408; -68.07221	North America			x		αβ
ME090	Métis	48.66408; -68.07221	North America			x		αβ
ME095	Métis	48.66408; -68.07221	North America	x	x	x		ββ
ME096	Métis	48.66408; -68.07221	North America			x		αβ
ME106	Métis	48.66408; -68.07221	North America	x	x	x		αβ
ME107	Métis	48.66408; -68.07221	North America			x		αβ
ME115	Métis	48.66408; -68.07221	North America			x		αβ
ME251	Métis	48.66408; -68.07221	North America			x		ββ
EO001	Osthassel	58.07068; 6.64346	Scandinavia	x	x		αα	αα
EO010	Osthassel	58.07068; 6.64346	Scandinavia				αβ	αβ
EO002	Osthassel	58.07068; 6.64346	Scandinavia	x	x	x	αβ	αβ
EO003	Osthassel	58.07068; 6.64346	Scandinavia				αβ	αβ
EO004	Osthassel	58.07068; 6.64346	Scandinavia				αβ	αβ
EO005	Osthassel	58.07068; 6.64346	Scandinavia	x	x	x	αα	αα

EO006	Osthassel	58.07068; 6.64346	Scandinavia	x	x	x	αα	αα
EO007	Osthassel	58.07068; 6.64346	Scandinavia				αβ	αβ
EO008	Osthassel	58.07068; 6.64346	Scandinavia				αα	αα
ESB01	Skadbergsand	58.4567; 5.91407	Scandinavia				ββ	ββ
ESB11	Skadbergsand	58.4567; 5.91407	Scandinavia				ββ	ββ
ESB12	Skadbergsand	58.4567; 5.91407	Scandinavia				ββ	ββ
ESB02	Skadbergsand	58.4567; 5.91407	Scandinavia				ββ	ββ
ESB03	Skadbergsand	58.4567; 5.91407	Scandinavia				ββ	ββ
ESB05	Skadbergsand	58.4567; 5.91407	Scandinavia				ββ	ββ
ESB06	Skadbergsand	58.4567; 5.91407	Scandinavia				ββ	ββ
ESB07	Skadbergsand	58.4567; 5.91407	Scandinavia				ββ	ββ
ESB08	Skadbergsand	58.4567; 5.91407	Scandinavia				ββ	ββ
ESB09	Skadbergsand	58.4567; 5.91407	Scandinavia				ββ	ββ
ESK01	Skeie	58.69733; 5.54083	Scandinavia	x	x	x	αβ	αβ
EY002	Ystad	55.425; 13.77254	Scandinavia	x	x	x	αβ	αβ
EY003	Ystad	55.425; 13.77254	Scandinavia	x	x	x		αα
EY004	Ystad	55.425; 13.77254	Scandinavia				αβ	αβ
EB16-SBS5	Skadbergsand	58.4567; 5.91407	Scandinavia				αβ	αβ
EB16-SBS6	Skadbergsand	58.4567; 5.91407	Scandinavia				αβ	αβ
EB16-SBS7	Skadbergsand	58.4567; 5.91407	Scandinavia				αβ	αβ
EB16-SBS8	Skadbergsand	58.4567; 5.91407	Scandinavia				αβ	αβ
EB16-SK1	Skeie	58.69733; 5.54083	Scandinavia				αβ	αβ
EB16-Y1	Ystad	55.425; 13.77254	Scandinavia				αβ	αβ
EB16-Y2	Ystad	55.425; 13.77254	Scandinavia				ββ	ββ
EB16-Y3	Ystad	55.425; 13.77254	Scandinavia				ββ	ββ
EB16-Y5	Ystad	55.425; 13.77254	Scandinavia				ββ	ββ
EB16-Y7	Ystad	55.425; 13.77254	Scandinavia				ββ	ββ
EB16-Y8	Ystad	55.425; 13.77254	Scandinavia				ββ	ββ
EB16-S1	Smyggahuk	55.337224; 13.36013	Scandinavia				αβ	αβ
EB16-S4	Smyggahuk	55.337224; 13.36013	Scandinavia				ββ	ββ
EB16-S5	Smyggahuk	55.337224; 13.36013	Scandinavia				ββ	ββ
EB16-S8	Smyggahuk	55.337224; 13.36013	Scandinavia				αβ	αβ
EB16-S9	Smyggahuk	55.337224; 13.36013	Scandinavia				αβ	αβ
EB16-S10	Smyggahuk	55.337224; 13.36013	Scandinavia				αβ	αβ

2- Inversion distribution & environmental variability in North America

Table S3: Environmental variables at sampled locations in North America

The “other seaweeds” category includes red, brown and green algae that did not belong to Fucaceae or Laminariceae

		GPS coordinates		Climatic and abiotic variables extracted from databases						Wrackbed characteristics			Wrackbed composition (%)				
	Location	Latitude (°)	Longitude (°)	Air T° (°C)	Precipitations (mm)	Sea T° (°C)	Sea Salinity (‰)	Tidal Amplitude (m)	Surface (m²)	Mean depth (m)	Mean T° (°C)	Salinity	Fucaceae	Laminariaceae	Plant Debris	Other Seaweeds	Zoosteraceae
**AG**	Anse du Griffon (QC)	48.93491	-64.30589	2.7	104.6	5.1	28.4	1.36	500	0.55	18.7	154	5	90	0	5	0
**BP**	Black Point (ME)	43.53059	-70.32209	8.1	113.9	8.8	31.5	3.06	10	0.2	16.2	8	25	5	0	20	50
**BS**	Blanc Sablon (QC)	51.41545	-57.15290	0.8	112.6	3.6	31.0	1.40	20	0.4	10.0		68	30	0	2	0
**BT**	Baie Trinité (QC)	49.41716	-67.30285	1.4	97.3	5.4	28.8	2.89	15	0.75	40.9	164	98	2	0	0	0
**CB**	Cow Bay (NS)	44.62190	-63.42112	6.4	140.9	7.1	30.3	1.46	90	0.35	17.1	10	5	70	0	25	0
**CE**	Cap Espoir (QC)	48.43087	-64.32778	3.4	109.6	6.1	29.8	1.12	2000	0.15	18.8	114	3	95	5	2	0
**CP**	Clam Point (NS)	44.72600	-62.91260	6.2	137.8	6.9	30.3	1.88	100	0.25	16.6	9	2	80	0	15	3
**GM**	Grands Méchins (QC)	49.00427	-66.97155	2.8	96.2	5.1	28.9	2.44	250	0.3	11.9	44	40	40	20	0	0
**HA**	Hampton (NH)	42.92098	-70.79826	8.7	114.4	9.3	31.4	2.87	500	0.45	37.3	179	50	2	0	50	0
**KA**	Kamouraska (QC)	47.56294	-69.87375	3.9	94.4	5.4	15.5	4.72	60	0.35	17.3	19	75	5	20	0	0
**MA**	Manomet Point (MA)	41.92654	-70.54451	9.8	119.9	10.0	31.8	3.13	10	0.15	12.0	9	90	2	0	2	5
**ME**	Métis (QC)	48.66408	-68.07221	2.4	93.0	4.4	24.6	3.06	300	0.3	19.4	95	60	0	14	1	25
**NB**	Naufrage Beach (PEI)	46.46795	-62.41561	5.7	109.2	8.3	30.0	0.73	2100	0.65	11.1	14	70	2	0	1	0
**PM**	Pabos Mills (QC)	48.31297	-64.69979	3.6	111.4	6.5	29.6	1.08	30	0.5	17.7	60	10	60	0	10	20
**RB**	Rivière du Bouleau (QC)	50.28161	-65.51516	1.3	98.9	4.9	30.2	1.53	0.2	0.2	17.0	80	80	20	0	0	0
**RC**	Rivière à Claude (QC)	49.22086	-65.89794	2.3	98.2	5.1	29.4	2.40	300	0.5	20.7	53	45	5	50	0	0
**RT**	Rivière du Tonnerre (QC)	50.28208	-64.61986	1.2	99.9	5.1	30.3	1.35	5	0.4	11.7	75	70	28	2	2	0
**SB**	Sally Beach (PEI)	46.25902	-62.37994	5.9	111.2	7.6	30.0	1.13	2100	0.5	12.8	15	99	0	0	1	0
**SI**	Saint Irénée (QC)	47.55973	-70.20425	2.7	105.1	5.4	15.5	4.72	250	0.15	6.8	0	80	15	0	5	0
**SS**	Saint Siméon (QC)	48.06991	-65.56586	4.2	103.3	7.0	29.1	1.66	200	0.2	14.9	113	20	50	0	5	25

Table S4: Sample genotyped, frequencies and H-W proportions

	Number of flies genotyped							Sex ratio (% of males)	Frequencies (corrected for sex-ratio 1:1)					Frequencies (corrected for natural sex-ratio)					Hardy-Weinberg proportions								
	Total	females			males														deviation in αα proportions			deviation in αβ proportions			deviation in ββ proportions		
		αα	αβ	ββ	αα	αβ	ββ		α	β	αα	αβ	ββ	α	β	αα	αβ	ββ		min CI	max CI		min CI	max CI		min CI	max CI
**AG**	111	4	36	14	3	30	24	34%	0.36	0.64	0.06	0.60	0.34	0.38	0.62	0.07	0.62	0.31	-51%	-81%	-22%	29%	12%	46%	-16%	-29%	-7%
**BP**	100	4	22	24	4	27	19	44%	0.33	0.68	0.08	0.49	0.43	0.32	0.68	0.08	0.48	0.44	-24%	-64%	15%	12%	-7%	30%	-6%	-16%	3%
**BS**	90	5	28	13	4	21	19	50%	0.37	0.63	0.10	0.54	0.36	0.37	0.63	0.10	0.54	0.36	-28%	-66%	4%	16%	-3%	37%	-10%	-23%	2%
**BT**	102	3	34	14	0	24	27	61%	0.31	0.69	0.03	0.57	0.40	0.30	0.70	0.02	0.55	0.43	-70%	-100%	-40%	32%	17%	46%	-15%	-24%	-7%
**CB**	117	9	35	14	5	34	20	44%	0.41	0.59	0.12	0.59	0.29	0.42	0.58	0.12	0.59	0.28	-30%	-57%	-6%	21%	5%	40%	-15%	-30%	-3%
**CE**	107	11	30	11	4	38	13	45%	0.46	0.54	0.14	0.63	0.22	0.46	0.54	0.15	0.63	0.22	-33%	-56%	-13%	28%	10%	46%	-24%	-42%	-8%
**CP**	96	6	30	12	6	18	24	34%	0.38	0.63	0.13	0.50	0.38	0.40	0.60	0.13	0.54	0.33	-11%	-45%	22%	7%	-13%	26%	-4%	-17%	8%
**GM**	106	9	38	3	3	45	8	21%	0.51	0.49	0.12	0.78	0.10	0.54	0.46	0.15	0.77	0.08	-56%	-75%	-38%	57%	42%	72%	-58%	-78%	-41%
**HA**	98	5	23	19	3	18	30	50%	0.29	0.71	0.08	0.42	0.50	0.29	0.71	0.08	0.42	0.50	-3%	-53%	48%	1%	-20%	19%	-1%	-8%	8%
**KA**	96	6	26	17	2	13	32	39%	0.28	0.72	0.08	0.40	0.51	0.31	0.69	0.09	0.43	0.48	2%	-52%	54%	-1%	-21%	20%	0%	-8%	9%
**MA**	96	7	23	16	4	29	17	49%	0.39	0.61	0.12	0.54	0.34	0.39	0.61	0.12	0.54	0.34	-23%	-55%	9%	14%	-6%	33%	-9%	-24%	3%
**ME**	102	6	41	6	1	35	13	64%	0.44	0.56	0.07	0.74	0.19	0.42	0.58	0.05	0.74	0.21	-65%	-87%	-44%	51%	34%	69%	-40%	-60%	-24%
**NB**	100	6	26	19	1	28	20	48%	0.34	0.66	0.07	0.54	0.39	0.34	0.66	0.07	0.54	0.39	-39%	-76%	-8%	20%	4%	39%	-10%	-22%	-2%
**PM**	95	3	26	18	4	21	23	38%	0.32	0.68	0.07	0.50	0.43	0.33	0.67	0.07	0.51	0.42	-29%	-73%	6%	13%	-3%	33%	-6%	-16%	2%
**RB**	98	2	31	17	3	28	17	28%	0.35	0.65	0.05	0.60	0.35	0.35	0.65	0.05	0.61	0.34	-59%	-89%	-29%	32%	16%	48%	-17%	-30%	-8%
**RC**	96	4	40	7	4	36	5	53%	0.48	0.52	0.08	0.79	0.12	0.48	0.52	0.08	0.79	0.12	-64%	-85%	-45%	59%	42%	74%	-54%	-73%	-36%
**RT**	89	5	29	28	1	13	13	21%	0.30	0.70	0.06	0.47	0.47	0.31	0.69	0.07	0.47	0.46	-34%	-82%	13%	14%	-5%	32%	-6%	-15%	2%
**SB**	95	4	18	24	6	22	21	38%	0.31	0.69	0.10	0.42	0.48	0.31	0.69	0.10	0.41	0.49	6%	-42%	47%	-3%	-21%	18%	1%	-8%	11%
**SI**	96	14	16	18	6	28	14	41%	0.44	0.56	0.21	0.46	0.33	0.44	0.56	0.22	0.44	0.34	9%	-16%	33%	-7%	-26%	12%	5%	-10%	21%
**SS**	98	13	24	11	7	28	15	54%	0.47	0.53	0.21	0.53	0.26	0.47	0.53	0.20	0.53	0.27	-7%	-32%	14%	7%	-13%	27%	-6%	-25%	11%
**Moy.**	99	6	29	15	4	27	19	43%	0.38	0.62	0.10	0.56	0.34	0.38	0.62	0.10	0.56	0.34	-30%			20%			-15%		
**Min.**	89	2	16	3	0	13	5	21%	0.28	0.49	0.03	0.40	0.10	0.29	0.46	0.02	0.41	0.08	-70%			-7%			-58%		
**Max.**	117	14	41	28	7	45	32	64%	0.51	0.72	0.21	0.79	0.51	0.54	0.71	0.22	0.79	0.50	9%			59%			5%		

Confidence intervals by bootstrapping: For each population, we simulated a set of karyotypes of the same sample size by sampling with replacement in a pool composed of the observed numbers of αα, αβ, ββ individuals. H-W deviations were calculated on the simulated set, this procedure was repeated 1000 times and the 2.5-97.5% quantiles on the distribution of bootstrapped values were taken as limits for the confidence interval.

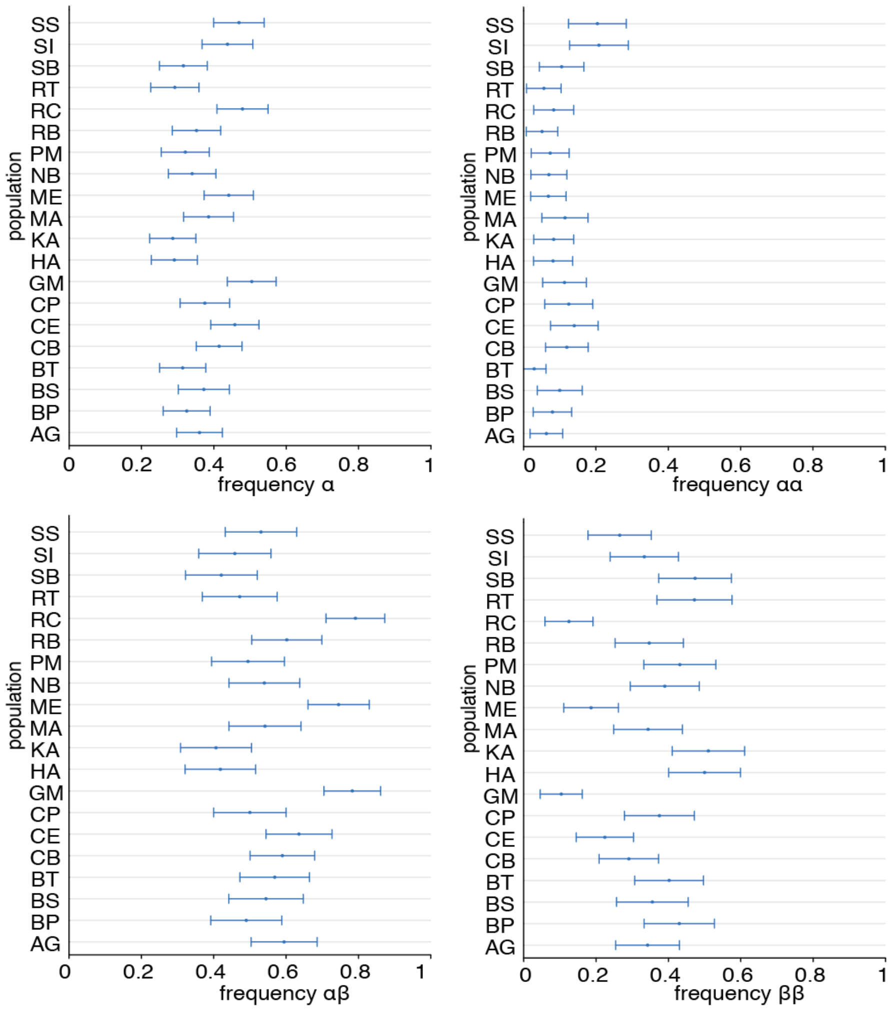

Fig S2: Heterogeneity of inversion & karyotype frequencies between populations. Deviance analysis and post-hoc contrasts of α rearrangement frequencies and each karyotype frequency between populations. Bars represent confidence intervals.

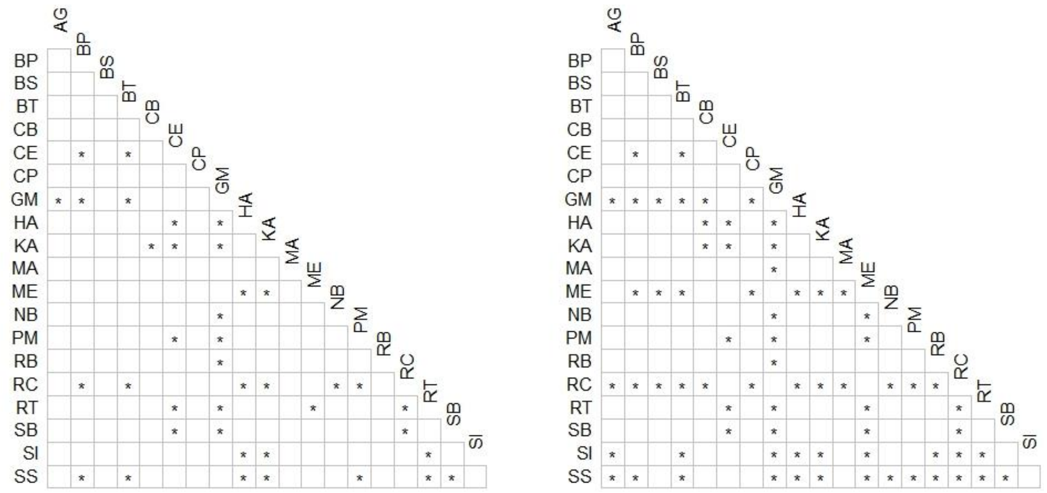

Fig S3: Heterogeneity of inversion frequency and karyotype proportions between populations. Pairwise comparison of inversion frequency (left) and karyotype proportions (right) between populations. Stars denote significantly different proportions under a chi² test, corrected for multiple test following (5)

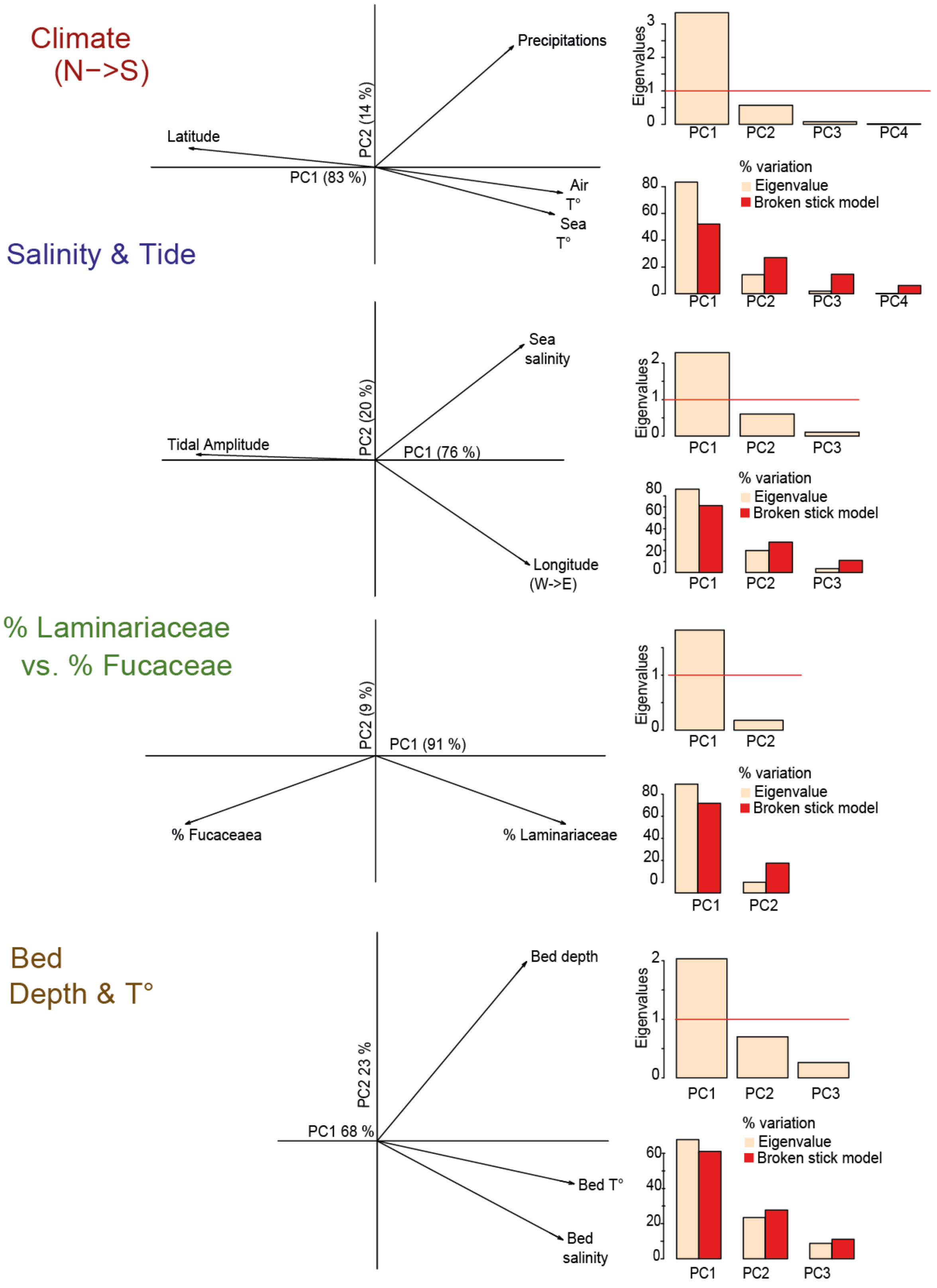

Fig S4: Summary variables drawn by PCA on environmental variables
For each summary variable (Fig. 3A), arrow plot representing the association between each environmental correlating variable and the first and second principal components (PC1, PC2). For all of them, the first PC was retained as the summary variable, following the Kaiser-Guttman and Broken Stick criteria (6). Eigen-values and percentage of variation explained by each PC are represented on the right side.

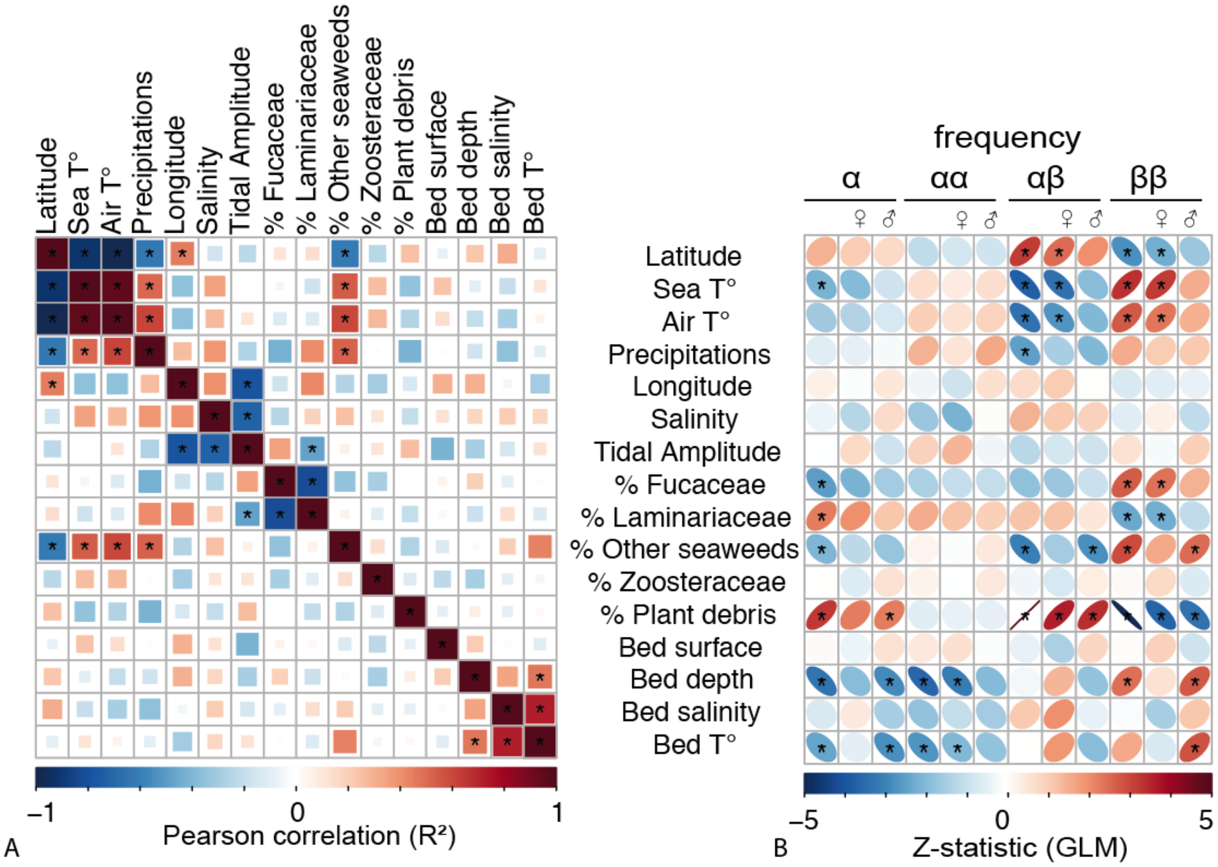

Fig S5: Association between environmental variables and inversion/karyotype frequencies
(A) Matrix of Pearson correlation between environmental variables. (B) Statistical association between each environmental predictor (alone) and the frequency of the α inversion rearrangement or the three karyotypes, for both sexes (ratio 1:1) or each sex independently. Colours and shape of the ellipse indicates the strength and direction of the statistical association while stars denote significance at 0.05 level, corrected for multiple comparison following (5)

Table S5: Best models of binomial logistic regression and beta-regression explaining and predicting inversion or karyotype frequencies by a combination of environmental variables
Analyses were conducted on frequencies corrected for sex-ratio 1:1, frequencies corrected for sex-ratio observed by sampling in natural populations, frequencies in male, frequencies in females.
Grey lines indicate a consensus model that minimized the AICc of both the betareg & GLM models.
%LF=% Laminariaceae vs. Fucaceae, %PD=% Plant Debris, %OS=% Other Seaweeds
F=females, M=males, SR 1:1=corrected for sex-ratio balanced, SR obs=corrected for sex-ratio observed in natural populations, freq=frequency

	freq	Predictors in the model	Beta-regression			GLM			R^2
			AICc	Δi	*WAICc*	AICc	Δi	*WAICc*	adjusted
F	α	~ %LF	-42	2	0.05	145	7	0.00	10%
F	α	~ %PD	-43	2	0.05	144	7	0.00	10%
F	α	~ %LF + %PD	-44	0	0.12	138	0	0.05	29%
F	α	~ %LF + %PD + Salinity&Tide	-42	2	0.04	138	0	0.05	34%
F	α	~ %LF + %PD + %OS	-42	3	0.03	138	1	0.04	33%
F	α	~ %LF + %PD + Climate	-41	3	0.03	139	2	0.03	31%
F	α	~ %LF + %PD + %OS + Salinity&Tide	-40	5	0.01	137	0	0.06	40%
M	α	~ Bed depth & T°	-43	0	0.08	141	7	0.00	13%
M	α	~ Bed depth & T° + %PD	-43	0	0.07	136	2	0.02	29%
M	α	~ Bed depth & T° + %PD + Salinity&Tide	-42	1	0.05	134	0	0.08	37%
M	α	~ Bed depth & T °+ %PD + %LF	-41	2	0.03	135	1	0.05	36%
SR 1:1	α	~ %PD	-48	1	0.07	179	15	0.00	14%
SR 1:1	α	~ %PD + Bed depth & T°	-48	1	0.05	173	9	0.00	24%
SR 1:1	α	~ %PD + %LF	-49	0	0.10	170	5	0.01	30%
SR 1:1	α	~ %PD + %LF + Bed depth & T°	-48	1	0.07	164	0	0.10	39%
SR 1:1	α	~ %PD + %LF + Bed depth & T° + %OS	-45	4	0.01	164	0	0.10	42%
SR 1:1	α	~ %PD + %LF + Bed depth & T° + Climate	-45	4	0.01	165	0	0.08	41%
SR obs	α	~ %PD	-48	1	0.07	179	15	0.00	14%
SR obs	α	~ %PD + %LF	-50	1	0.11	167	8	0.00	37%
SR obs	α	~ %PD + %LF + Bed depth & T°	-51	0	0.17	159	0	0.11	50%
SR obs	α	~ %PD + %LF + Bed depth & T° + %OS	-48	3	0.04	159	0	0.10	53%
SR obs	α	~ %PD + %LF + Bed depth & T° + Climate	-48	3	0.03	159	0	0.10	52%
F	αα	~ Bed depth & T°	-54	0	0.11	101	4	0.02	18%
F	αα	~ Bed depth & T° + Salinity&Tide	-53	2	0.05	101	4	0.01	24%
F	αα	~ Bed depth & T° + Salinity&Tide + %LF	-52	3	0.03	99	0	0.06	36%
M	αα	~ Bed depth & T°	-82	2	0.07	78	0	0.05	23%
M	αα	~ Bed depth & T° + %LF	-84	0	0.20	78	0	0.05	34%
SR 1:1	αα	~ Bed depth & T°	-73	0	0.15	110	2	0.02	28%
SR 1:1	αα	~ Bed depth & T° + %LF	-72	0	0.13	108	0	0.05	37%
SR 1:1	αα	~ Bed depth & T° + Salinity&Tide + %LF	-70	2	0.05	108	0	0.05	43%
SR obs	αα	~ Bed depth & T°	-71	1	0.11	114	4	0.01	34%
SR obs	αα	~ Bed depth & T° + %LF	-72	0	0.17	111	0	0.05	45%
SR obs	αα	~ Bed depth & T° + Salinity&Tide + %LF	-70	2	0.07	111	0	0.06	51%

	freq	Predictors in the model	Beta-regression			GLM			R²
			AICc	Δi	*wAICc*	AICc	Δi	*wAICc*	adjusted
F	αβ	~ %PD + Bed depth & T°	-29	0	0.05	125	7	0.00	41%
F	αβ	~ %PD + Bed depth & T° +Salinity&Tide	-30	0	0.06	122	3	0.02	52%
F	αβ	~ %PD + Bed depth & T° + %LF	-30	0	0.06	122	3	0.02	52%
M	αβ	~ %PD	-22	0	0.14	146	4	0.00	20%
M	αβ	~ %PD + %OS	-21	2	0.06	144	2	0.01	26%
M	αβ	~ %PD + Salinity&Tide	-21	2	0.06	144	2	0.01	25%
M	αβ	~ %PD + Salinity&Tide + Bed depth & T°	-19	4	0.02	142	0	0.03	30%
M	αβ	~ %PD + %OS + %LF	-18	4	0.02	143	0	0.03	30%
M	αβ	~ %PD + %OS + Salinity&Tide	-18	4	0.02	143	0	0.03	30%
SR 1:1	αβ	~ %PD	-34	0	0.12	167	10	0.00	34%
SR 1:1	αβ	~ %PD + Salinity&Tide	-34	0	0.13	159	2	0.02	45%
SR 1:1	αβ	~ %PD + %LF	-33	1	0.07	163	6	0.00	40%
SR 1:1	αβ	~ %PD + Salinity&Tide + Climate	-31	3	0.04	158	1	0.03	48%
SR 1:1	αβ	~ %PD + Salinity&Tide + Climate + %LF	-28	6	0.01	157	0	0.04	50%
SR 1:1	αβ	~ %PD + Salinity&Tide + %OS + %LF	-28	6	0.01	157	0	0.05	50%
SR obs	αβ	~ %PD	-35	1	0.09	165	14	0.00	35%
SR obs	αβ	~ %PD + Salinity&Tide	-36	0	0.13	154	4	0.01	48%
SR obs	αβ	~ %PD + %LF	-35	1	0.10	157	6	0.00	45%
SR obs	αβ	~ %PD + Salinity&Tide + %LF	-34	2	0.05	152	2	0.03	52%
SR obs	αβ	~ %PD + Salinity&Tide + %LF + %OS	-31	4	0.01	151	0	0.07	56%
SR obs	αβ	~ %PD + Salinity&Tide + %LF + Climate	-31	5	0.01	151	1	0.05	55%
F	ββ	~ %PD	-28	1	0.09	136	10	0.00	28%
F	ββ	~ %PD + %LF	-30	0	0.19	127	0	0.05	45%
F	ββ	~ %PD + %LF + Climate	-28	2	0.07	126	0	0.06	49%
F	ββ	~ %PD + %LF + %OS	-27	3	0.05	127	0	0.05	48%
M	ββ	~ %PD	-21	0	0.12	153	12	0.00	15%
M	ββ	~ %PD + Bed depth & T°	-21	0	0.11	146	5	0.01	27%
M	ββ	~ %PD + Bed depth & T° + Salinity&Tide	-19	2	0.04	141	0	0.08	33%
SR 1:1	ββ	~ %PD	-30	0	0.13	187	17	0.00	26%
SR 1:1	ββ	~ %PD + %LF	-31	0	0.15	175	5	0.01	38%
SR 1:1	ββ	~ %PD + %LF + %OS	-29	2	0.07	170	0	0.13	44%
SR obs	ββ	~ %PD + %LF	-32	0	0.22	168	5	0.01	46%
SR obs	ββ	~ %PD + %LF + %OS	-31	1	0.11	163	0	0.12	53%
SR obs	ββ	~ %PD + %LF + %OS + Bed depth & T°	-27	5	0.02	164	1	0.08	54%

Salinity/tidal amplitude was a marginal predictor of the karyotype proportions (9%(αβ), 6%(αα), 1%(ββ) of variance,). Yet, it was not associated to variation in α frequency, possibly because the decrease in αβ proportions in the inner estuary was balanced by an increase in both homokaryotypes (Fig.3B). Wrackbed surface or the proportion of Zoosteraceae were never retained in the best models (Table.S5-7).

Table S6: Dirichlet regression between karyotype composition and environmental variables. Only best models are presented. These were selected based on AICc, parsimony (lesser number of predictors), and fit between observed and predicted values (R^2 adjusted), as detailed in the first column. %LF=% Laminariaceae vs. Fucaceae, %PD=% Plant Debris, %OS=% Other Seaweeds

DIRICHLET REGRESSION

	Predictors	*AICc*	*ΔAICc*	*R^2 αα*	*R^2 αβ*	*R^2 ββ*	*mean R^2*
Best models based on AICc	%OS + %LF + BedDepth&T°	-97	**0**	-7%	1%	-2%	-3%
	%PD	-94	**3**	-5%	**34%**	**26%**	18%
	%OS	-92	**5**	-5%	7%	5%	2%
Best models based on mean R^2 (keeping ΔAICc below 50)	%PD + BedDepth&T° + %LF	-58	39	22%	32%	31%	**28%**
	%PD + BedDepth&T° + Salinity&Tide	-55	41	15%	36%	24%	**25%**
	%PD + BedDepth&T°	-84	13	18%	31%	25%	**25%**
Most parcimonious models best predicting αα proportions	BedDepth&T° + %LF	-66	31	**32%**	-6%	-2%	8%
	BedDepth&T°	-80	16	**28%**	5%	-3%	10%
Most parcimonious models best predicting αβ proportions	%PD + Salinity&Tide	-82	14	-11%	**38%**	25%	17%
	%PD + %LF	-83	14	-9%	**36%**	33%	20%
	%PD	-94	3	-5%	**34%**	26%	18%
Most parcimonious models best predicting ββ proportions	%PD + %LF	-83	14	-9%	36%	**33%**	20%
	%PD	-94	3	-5%	34%	**26%**	18%

Table S7: Best models of redundancy analysis including spatial autocorrelation

In complement, models of redundancy analysis were build including environmental predictors identified as relevant and, to control for spatial auto-correlation, spatial variables. Those spatial variables describe geographic proximity between populations based on Principal Coordinates of Neighbourhood Matrix (PCNM) map distances (7). Best models were selected using a stepwise backward model selection using permutation tests (8) and mostly support a significant role of the composition of the wrackbed in explaining variations of frequency between populations.

Spatial variables were not a significant factor except in one model addressing variation in αα frequency. When controlling for this spatial autocorrelation, the best environmental predictor (depth/T° of the wrackbed) still explained 18% of the variance in αα frequency.

**Frequency**	**RDA best model**	**R^2 adjusted**
α	~ %PD + %LF	25%
αα	~ Bed depth & T° [corrected by spatial autocorrelation]	25% [18%]
αβ	~ %PD	25%
ββ	~ %PD + %LF	35%

%LF=% Laminariaceae vs. Fucaceae, %PD=% Plant Debris, %OS=% Other Seaweeds

3- Size measurement and analysis

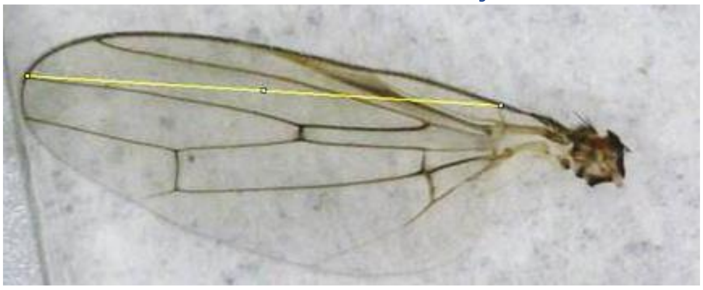

Fig S6: Wing length measurement

Left wing was dissected and placed between a slide and a slip cover with a drop of 96% ethanol, and then photographed with an Olympus DP20 camera mounted on the magnifier, controlling for scale. Wing length was recorded in ImageJ as the distance between the distal side of the humeral break at the posterior end of the coastal cell and the distal tip of the longitudinal vein III, as shown here by the yellow line.

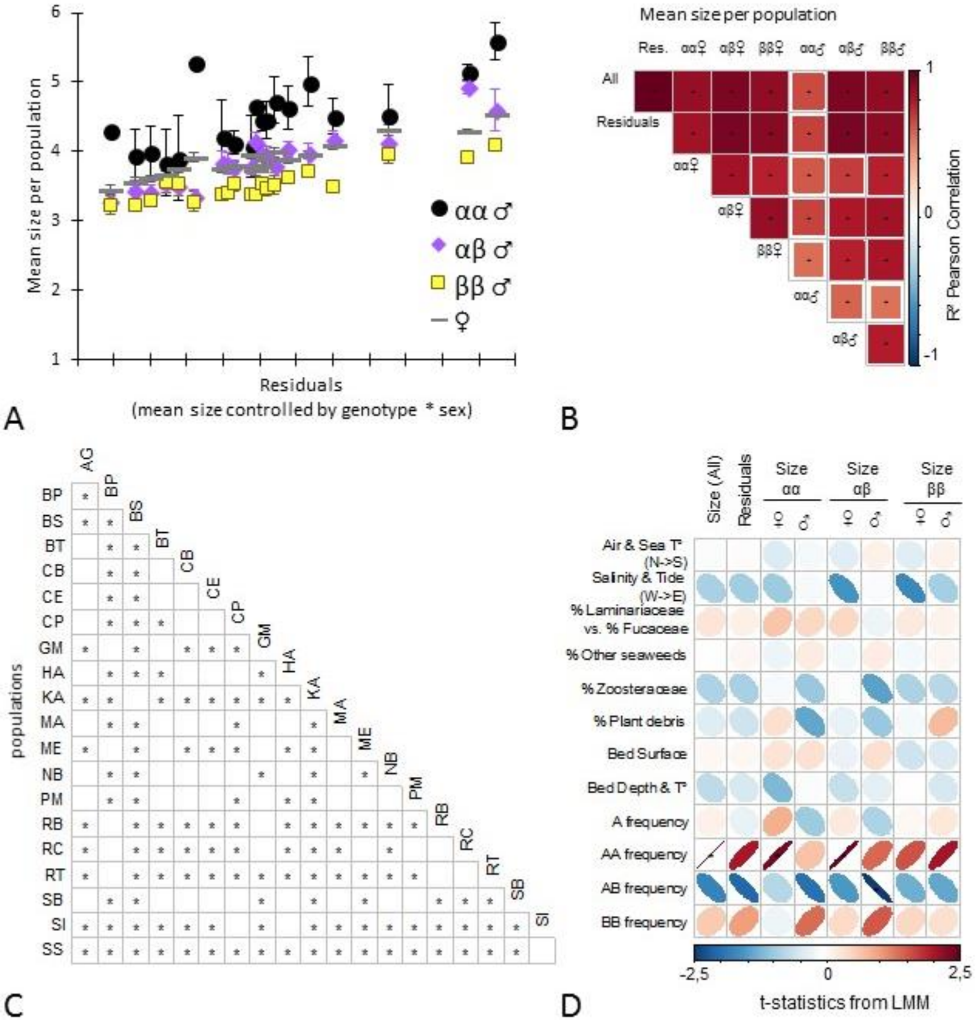

Fig S7: Size variation between populations in relation with local factors.

(A) Mean size for sex*karyotype groups per population. (B) Matrix of Pearson correlation between mean size per population. Stars denote significance at 0.05 level for correlation test. (C) Pairwise comparison of size (residuals, controlled by karyotype*sex) between populations. Stars denote significance at 0.05 level for the pairwise t-test (correction B&H). (D) Statistical association between size and each environmental predictor or the frequency of the inversion/ of the karyotypes. Colours and shape of the ellipse indicates the strength and direction of the statistical association. Stars denote significance at 0.05 level in a linear mixed model (LMM).

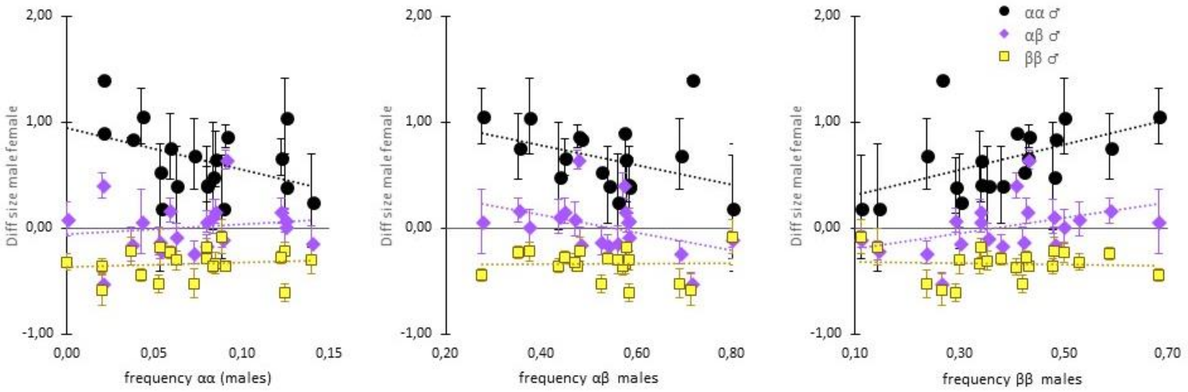

Fig S8: Difference between average male and female wing size at each population as a function of karyotype frequency.

Dots represent the average by male karyotype, bars the standard error of the mean, and dotted lines represent Pearson's correlations.

4- Comparison between North American and European populations

Methods for comparison North America/Europe

Karyotype frequencies and wrackbed composition were reported by Day et al (9) for 13 Scandinavian populations (Table S8). Using locality names, we inferred approximate GPS coordinates and extracted the same large-scale climatic/abiotic variables as for North America (Table S8). For tidal amplitude, we used archives from The Norwegian Hydrographic Service and data from Gilburn & Day, 1994 (10). In the original publication, seaweed composition is reported with stars as an indicator of presence and abundance (ranging from 0 stars to 4 stars) and we interpreted those data as a ranked variable describing variation in the abundance of each seaweed between populations.

For comparison, the association between inversion/karyotype frequency and each raw environmental variable was analysed as in North America with a binomial GLM and a logit link (Fig.S10). Yet, given the high correlation between all variables in Scandinavia, we applied a PCA on all the variables and build multi-variable modelling on the first, second, third and fourth PCs (summary variables describing a total of 97% of environmental variance).

Table S8: Environmental variables at locations sampled by Day *et al* (1983) (9) in Scandinavia

		GPS coordinates		Climatic and abiotic variables extracted from databases					Wrackbed composition				Frequency			
	Location	Latitude (°)	Longitude (°)	Air T° (°C)	Precipitations (mm)	Sea T° (°C)	Sea Salinity (‰)	Tidal Amplitude (m)	Fucaceae	Laminariaceae	Other Seaweeds	Zoosteraceae	α	αα	αβ	ββ
**YST**	Ysterbröd	58.4355	5.9066	9.8	150.3	7.1	33	0.4	2	2	0	0	0.46	0.13	0.65	0.22
**ELL**	Ellestrand	58.17735	6.67398	9.6	162.8	7.0	33	0.4	0	4	0	0	0.47	0.15	0.63	0.22
**OST**	Østhasselstrand	58.07261	6.64787	10.1	155.8	7.5	33	0.4	0	4	0	0	0.45	0.15	0.6	0.24
**HOM**	Homborsund	58.26987	8.52025	10.0	115.0	7.4	31	0.4	2	2	0	0	0.44	0.16	0.56	0.28
**BUA**	Bua	57.23683	12.12214	8.9	77.2	7.7	19	0.3	4	0	0	0	0.38	0.08	0.61	0.31
**FOR**	Fornaes fyr	56.44341	10.95731	10.0	57.5	7.9	22	0.3	4	0	0	0	0.41	0.07	0.67	0.26
**TRA**	Träslövsläge	57.06118	12.28283	9.7	76.9	7.7	18	0.3	2	0	2	0	0.39	0.07	0.64	0.29
**STE**	Steningestrand	56.75795	12.63745	9.8	77.5	7.6	16	0.2	4	0	0	0	0.48	0.19	0.59	0.23
**TOR**	Torekov	56.42363	12.6273	9.8	72.7	7.6	15	0.2	2	0	2	0	0.4	0.11	0.57	0.31
**VEJ**	Vejbystrand	56.31588	12.76943	9.8	72.6	7.6	15	0.2	3	0	1	0	0.5	0.16	0.67	0.17
**MOL**	Mölle	56.28219	12.50212	9.8	67.7	7.7	15	0.2	3	0	1	0	0.42	0.16	0.53	0.31
**VIK**	Viken	56.14592	12.58042	9.7	66.4	7.8	15	0.2	4	0	0	0	0.46	0.12	0.69	0.19
**KAM**	Kämpinge	55.40581	12.97699	9.5	57.9	8.2	13	0.1	1	0	0	3	0.35	0.06	0.58	0.37
**SKA**	Skateholm	55.37896	13.4726	9.3	62.1	8.0	11	0.1	2	0	2	0	0.35	0.09	0.51	0.4
**KAS**	Kåseberga	55.38655	14.06511	9.3	58.0	8.0	10	0.1	1	0	3	0	0.35	0.09	0.51	0.39

Results of the comparison North America/Europe

Inversion distribution and karyotype frequencies as reported by Day *et al* (7) mirrored the pattern that we observed in American populations since all investigated populations in Scandinavia were also polymorphic with a slightly less frequent α rearrangement (mean: 42% [35-50%]), significant heterogeneity between populations (deviance =77.4, df=14, p<0.001) and significant heterokaryotype excess (+24% on average, Fig.1).

Multiple ecological gradients (climate, salinity, tidal amplitude, and seaweed composition) correlated along the Scandinavian cline (Fig.S9D). For instance, Laminariaceae were more abundant in the norther part of the cline and other seaweeds in the southern part. Therefore, disentangling which ecological dimensions that underlined inversion frequency and karyotype composition in Europe was not possible. We therefore limited our comparison to the parallelism in the direction of the association between the inversion frequency and environmental variables.

Parallelism in the direction of association between inversion/karyotype frequencies and environment was observed for two major predictors, namely the North-South thermic cline and the seaweed composition of the wrackbed in Laminariaceae and other seaweeds. On both continents, the α frequency decreased along the North-South cline (negatively associated with latitude) with warmer air temperature observed in the south (Fig.S9BC). This is linked to significantly higher proportions of the karyotype ββ at southern warmer locations, at the depends of αβ in North America and at the depends of αα in Scandinavia. Both in America and Scandinavia, higher abundance of Laminariaceae was positively associated with α frequency linked to higher proportions of αα relatively to ββ. Similarly, higher abundance of other seaweeds was negatively associated with α frequency linked with higher ββ proportions relatively to αβ (Fig.S9BC). The abundance of Fucaceae was not a relevant predictor of α frequency in Scandinavia and showed no parallelism. Other climatic descriptors, such as precipitations and sea temperature, did not correlate with the North-South cline in the same direction on both continents, which may explain why they were differently associated with α frequency and suggest that parallelism is rather linked either to air temperature or other seaweeds abundance. Salinity and tidal amplitude were not significantly associated with α frequency in North America, while in Scandinavia they covaried along the cline, and as such, were also associated with variation in α frequency.

Overall, the cline was a much more important predictor of α frequency in Scandinavia, explaining up to 30% of the variation (PC1, Fig.S11, Table S9), likely because all factors co-varied along this gradient. Yet, seaweed composition of the wrackbed was an additional predictor of α frequency in Scandinavia, explaining 15% of variance (PC2, Fig. S11, Table S9), and underlying the relative proportions of ββ vs. αβ (Table S9-10).

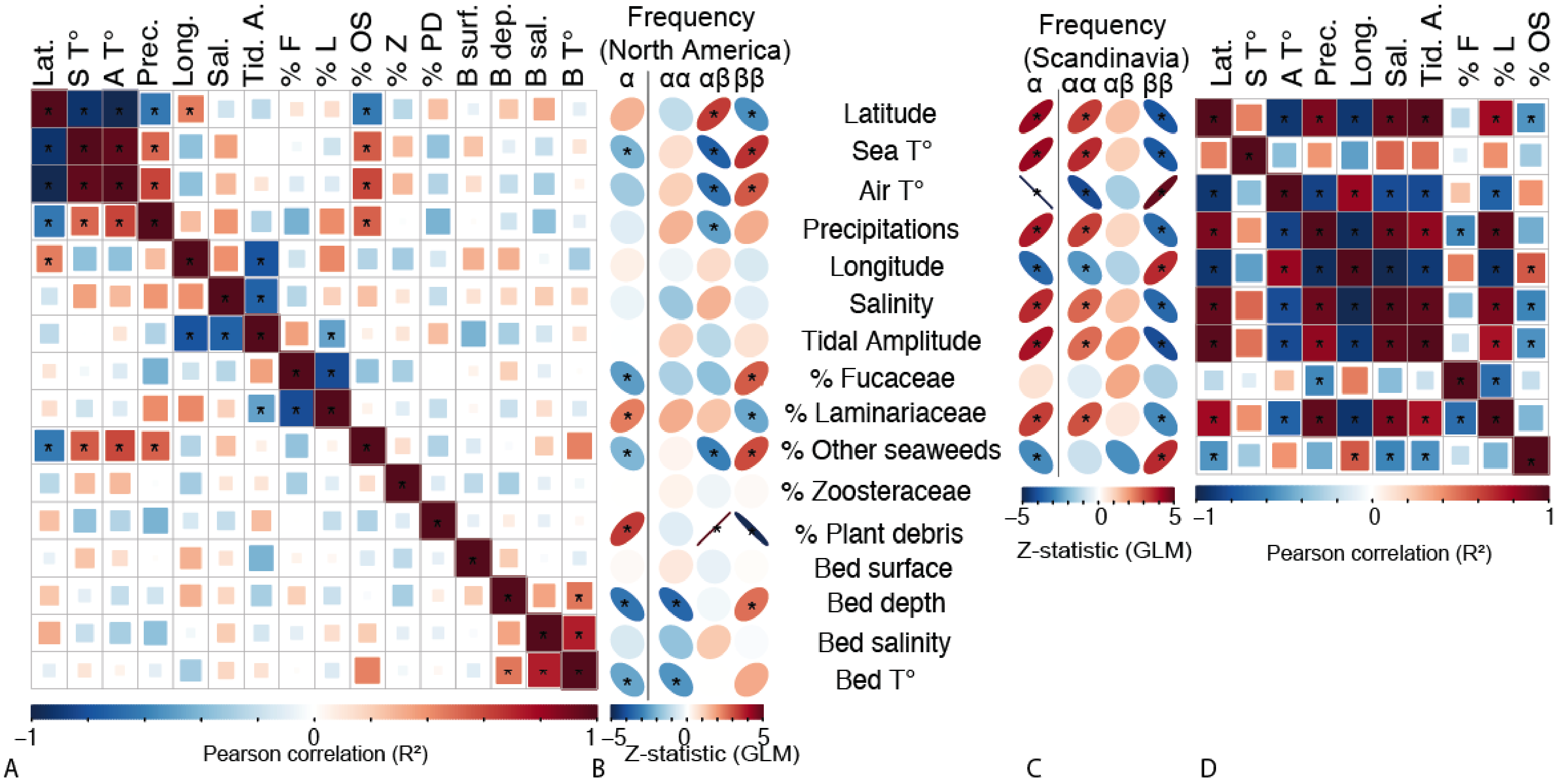

Fig S9: Comparing parallelism in the directionality of association between inversion/karyotype frequency and environmental variables
(A) Matrix of Pearson's correlation between environmental variables in North America (B) Statistical associations between each environmental predictor and the frequency of α rearrangement or each karyotype in North America. (C) Statistical associations between each environmental predictor and the frequency of α rearrangement or each karyotype in Scandinavia. (D) Matrix of Pearson's correlation between environmental variables in Scandinavia. Strength and direction of the statistical association (GLM) are indicated by the shape of the ellipse and its colour (red: positive, blue: negative). Stars denote significance at 0.05 level , corrected for multiple comparison following (5)

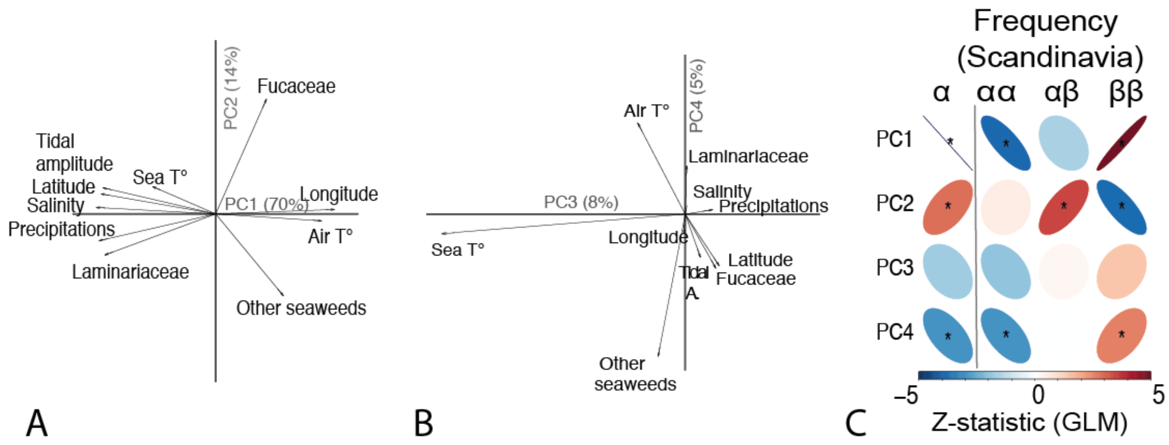

Fig S10: PCA on environmental variables in Scandinavia and association with inversion and karyotype frequencies

(A-B) PCA on all environmental variables correlating along the Scandinavian cline. The plot represents the association between each environmental variable and each PC retained for the analysis. (C) Statistical association between PCs and the frequency of the α inversion rearrangement or the three karyotypes. Colours and shape of the ellipse indicates the strength and direction of the statistical association while stars denote significance at 0.05 level.

Table S9: Best models explaining and predicting inversion or karyotype frequencies in Scandinavia by a combination of environmental variables

Grey lines indicate a consensus most plausible model minimizing the AICc of both the betareg & GLM models. %LF=% Laminariaceae vs. Fucaceae, %OS=% Other Seaweeds (Ceramium), cline NS= summary variable for abiotic-climatic variation along the North-South cline

	**Beta-regression**			**GLM**			**R^2**	**Jack-knife**
**Model**	**AICc**	**Δi**	***WAICc***	**AICc**	**Δi**	***WAICc***	**adjusted**	**Diff (obs-predict)**
frequency α ~ PC1 +PC2	-47	0	0.39	150	3	0.09	42%	3%
frequency α ~ PC1	-46	1	0.25	159	13	0.00	27%	3%
frequency αα ~ PC1	-50	0	0.44	132	7	0.01	14%	3%
frequency αβ ~ PC2	-41	0	0.45	137	2	0.14	25%	5%
frequency αβ ~ PC1 + PC2	-40	2	0.21	135	0	0.36	30%	4%
frequency ββ ~ PC1 + PC2	-38	0	0.50	143	0	0.25	50%	4%

Table S10: Best Dirichlet regression models explaining and predicting karyotype composition in Scandinavia by a combination of environmental variables transformed with PCA (cf Fig. S12)

Predictors	***AICc***	***ΔAICc***	***R^2 αα***	***R^2 αβ***	***R^2 ββ***	***mean R^2***
PC1	-89.9	0	**14%**	7%	25%	15%
PC2	-78.7	11.2	-4%	**25%**	22%	14%
PC1 + PC2	-71	18.9	8%	25%	**43%**	**25%**

References

1. Butlin R, Collins P, Skevington S, Day T. Genetic variation at the alcohol dehydrogenase locus in natural populations of the seaweed fly, *Coelopa frigida*. Heredity. 1982;48(Pt 1):45–55.

2. Aljanabi SM, Martinez I. Universal and rapid salt-extraction of high quality genomic DNA for PCR-based techniques. Nucleic Acids Res. 1997;25(22):4692–3.

3. Olsen JB, Wenburg J, Bentzen P. Semiautomated multilocus genotyping of Pacific salmon (Oncorhnychus spp.) using microsatellites. Mol Mar Biol Biotechnol. 1996;5(4):259–72.

4. Wellenreuther M, Rosenquist H, Jaksons P, Larson KW. Local adaptation along an environmental cline in a species with an inversion polymorphism. J Evol Biol. 2017;30(6):1068–77.

5. Benjamini Y, Hochberg Y. Controlling the false discovery rate: a practical and powerful approach to multiple testing. J R Stat Soc Ser B Methodol. 1995;289–300.

6. Borcard D, Gillet F, Legendre P. Numerical ecology with R. Springer Science & Business Media; 2011.

7. Borcard D, Legendre P. All-scale spatial analysis of ecological data by means of principal coordinates of neighbour matrices. Ecol Model. 2002;153(1):51–68.

8. Blanchet FG, Legendre P, Borcard D. Forward selection of explanatory variables. Ecology. 2008;89(9):2623–32.

9. Day T, Dawe C, Dobson T, Hillier P. A chromosomal inversion polymorphism in Scandinavian populations of the seaweed fly, *Coelopa frigida*. Hereditas. 1983;99(1):135–45.

10. Gilburn AS, Day TH. Sexual Dimorphism, Sexual Selection and the αβ Chromosomal Inversion Polymorphism in the Seaweed Fly, *Coelopa frigida*. Proc R Soc Lond B Biol Sci. 1994;257(1350):303–9.
